# Preheparin Serum Lipoprotein Lipase Mass as a Coronary Risk Factor in Patients With Chronic Kidney Disease

**DOI:** 10.14740/cr2192

**Published:** 2026-02-28

**Authors:** Takashi Hitsumoto

**Affiliations:** Hitsumoto Medical Clinic, Shimonoseki City, Yamaguchi 750-0025, Japan. Email: thitsu@jcom.home.ne.jp

**Keywords:** Preheparin serum lipoprotein lipase mass, Chronic kidney disease, Primary coronary artery disease event, Prospective study, Insulin resistance, Advanced glycation end products, Inflammation

## Abstract

**Background:**

A significant association between lower preheparin serum lipoprotein lipase mass (pre-LpL mass) and coronary artery disease (CAD) has been reported in several clinical studies. However, the predictor of a pre-LpL mass as a CAD event in patients with chronic kidney disease (CKD) remains unclear. This prospective study aimed to investigate the clinical significance of a pre-LpL mass as a predictor of primary CAD events in patients with CKD.

**Methods:**

A total of 480 CKD patients who did not develop CAD among outpatients who visited the clinic were enrolled. Using receiver operating characteristic curve analysis for a primary CAD event, participants were divided into two groups (low pre-LpL mass (group L, n = 211) or high pre-LpL mass (group H, n = 269)) by pre-LpL mass, and significance of a pre-LpL mass as a predictor for the primary CAD events was performed.

**Results:**

At baseline, skin autofluorescence, an indicator of advanced glycation end products *in vivo*, and high-sensitivity C-reactive protein (hs-CRP) concentration, an indicator of inflammation, were significantly higher in group L than in group H. During the median observation period of 107 months, 42 patients experienced a CAD event (group L: n = 31 (14.7%) vs. group H: n = 11 (4.1%)). Group L had a significantly higher incidence of primary CAD events than group H (P < 0.001, log-rank test). Furthermore, patients in group L were at a significantly higher risk of developing a primary CAD event than those in group H based on the multivariate Cox regression analysis (hazard ratio: 2.80; 95% confidence interval, 1.39–5.64; P = 0.003). However, skin autofluorescence and hs-CRP were also significant factors for a primary CAD event.

**Conclusions:**

The prospective study showed that a decrease in pre-LpL mass is a useful predictor of a primary CAD event in patients with CKD. Additionally, background factors such as an increase in advanced glycation end products and inflammation are also an important factor in these patients.

## Introduction

Chronic kidney disease (CKD) is one of the major risk factors for the development of coronary artery disease (CAD) as reported in many clinical studies [1–3]. Furthermore, patients with CKD who develop CAD present with a much worse prognosis than non-CKD patients [4, 5]. In addition to a decrease in the kidney function, several clinical factors have been cited as risk factors of a CAD event in patients with CKD [6–8]. However, even with the management of these risk factors, residual risks still exist. Therefore, it is considered useful to validate the discovery of novel CAD risk factors, including biomarkers, for the prevention of CAD in CKD patients through prospective study.

Lipoprotein lipase (LpL) is an enzyme that has a central role in the hydrolysis of triglycerides; moreover, it has been reported that a decrease in this enzyme’s activity promotes the appearance of arteriosclerosis-induced lipoproteins such as remnant lipoproteins and small-sized-particle low-density lipoprotein [9]. Conventionally, LpL function is evaluated through the measurement of its activity and amount of protein after intravenous heparin injection; however, with the advent of a sensitive method of measuring the amount of LpL protein, measuring the amount of LpL protein present in serum prior to intravenous heparin injection (preheparin serum lipoprotein lipase mass (pre-LpL mass)) has become possible [10]. Clinical studies have shown that low pre-LpL mass is related to the pathophysiology of metabolic syndrome such as insulin resistance, low serum adiponectin concentration, visceral fat accumulation, and oxidative stress [11, 12]. Furthermore, researchers have reported that a low pre-LpL mass is significantly associated with the presence of CAD [13–17]. Several reports indicated a relationship between a decrease in LpL activity and CKD [18, 19], suggesting that the low pre-LpL mass is also related to coronary arteriosclerosis progression in CKD patients. However, the clinical significance of pre-LpL mass as a CAD risk factor in patients with CKD remains unclear. It would be clinically relevant if the pre-LpL mass can be a novel predictor of CAD event in patients with CKD. In this prospective study, the author examined the clinical significance and possibility of the pre-LpL mass as a predictor of primary CAD events in patients with CKD.

## Materials and Methods

### Patients

This study was performed in a single-center unit. A total of 506 consecutive CKD patients with no history of CAD who visited Hitsumoto Medical Clinic in Yamaguchi Prefecture between July 2015, and June 2017 were initially enrolled. Of these, six patients who lacked informed consent and 20 patients who lacked the clinical data including pre-LpL mass required for this study were excluded. Ultimately, 480 patients (160 men (33.3%), 320 women (66.7%)) were enrolled in the study. The estimated glomerular filtration rate (eGFR) as a maker of kidney function was calculated using a formula developed for the Japanese population [20], and eGFR levels < 60 mL/min/1.73 m^2^ was defined as CKD. No patients on dialysis were observed in this study.

### Ethical considerations

This study was conducted in accordance with the Declaration of Helsinki guidelines. In this study, the informed consent was obtained by all patients, and the study protocol was approved by the Institutional Review Board of Hitsumoto Medical Clinic (date of approval: June 15, 2015; approval number: HMC-2015-6).

### Pre-LpL mass measurement

The pre-LpL mass was measured through a sandwich enzyme-linked immunosorbent assay using a specific monoclonal antibody against LpL via a commercial device (Daiichi Pure Chemicals, Japan), as described by previous reports [10]. The linearity of this assay system was observed between 5 and 400 ng/mL. The within-run coefficient of variation was 2.8%, and the between-day coefficient of variation for this assay was 4.3%, which has been described in a previous report [15], indicating reliability.

### Evaluation of clinical parameters

The body mass index was used to evaluate the degree of obesity. The definition of a smoker was determined from the medical record information and was defined as patients who smoked at the baseline. Hypertension, dyslipidemia, and diabetes mellitus were described in previous reports [21]. Blood and urine data were measured on a fasting state. Blood and urine data comprised of the following: serum creatinine concentration, serum lipid concentration, blood glucose concentration, serum high-sensitivity C-reactive protein (hs-CRP) concentration, and urinary albumin concentration. Serum creatinine concentration, age, and sex were used to calculate the eGFR [20], and albuminuria was defined as having a urinary albumin concentration of 30 mg/g Cr or more. The triglyceride-glucose (TyG) index was used as a marker of insulin resistance by an existing report (Ln (fasting serum triglyceride levels (mg/dL) × fasting blood glucose levels (mg/dL)/2)) [22]. Skin autofluorescence (AF), a marker of advanced glycation end products (AGEs) *in vivo*, was measured on the forearm of patients using commercial devices (AGE Reader; DiagnOptics, Groningen, Netherlands) in previous reports [23, 24]. Oral medications, such as renin–angiotensin system (RAS) inhibitor and statin, were also investigated.

### Determination of a CAD event and grouping

**Figure 1:**
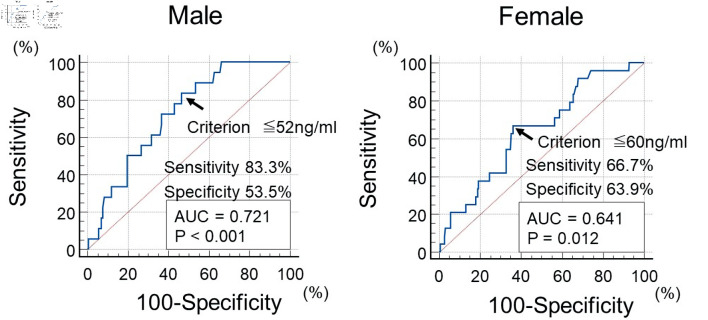
The optimal cut-off value of pre-LpL mass for initial CAD event using ROC curve analysis. Using ROC curve analysis, participants were divided into two groups (low pre-LpL mass (group L) or high pre-LpL mass (group H)) by pre-LpL mass (cut-off value: 52 ng/mL for men, 60 ng/mL for women). The arrows indicate the optimal cut-off point value. CAD: coronary artery disease, pre-LpL mass: preheparin serum lipoprotein lipase mass; ROC: receiver operating characteristic; AUC: area under the curve.

This study examined whether the enrolled patients developed CAD during the observation period until June 2025 using medical records. A primary CAD event was defined as patients who underwent coronary revascularization and/or medication for angina pectoris or patients who developed acute myocardial infarction. Existing reports have indicated that pre-LpL mass values differ by gender (being lower in male compared to female) in CAD patients, and the cut-off values were determined based on this gender difference in this study [25]. Patients were assigned into two groups according to the optimal cut-off value of the pre-LpL mass (male: 52 ng/mL; female: 60 ng/mL), which was estimated using the receiver operating characteristic curve analysis for a CAD event (Fig. 1): low (group L, n = 211) or high (group H, n = 269).

### Statistical analysis

The statistical analysis was conducted using the commercialized software Stat View-J 5.0 (HULINKS Inc., Tokyo, Japan) and MedCalc (MedCalc Software, Ostend, Belgium). The optimal cut-off value of the pre-LpL mass for a CAD event was determined using the receiver operating characteristic curves via the Youden index [26]. Continuous variables were expressed as means and standard deviations or median (interquartile range). To perform comparisons between groups, an unpaired *t*-test or Mann–Whitney U test was used. A Kaplan–Meier analysis was performed to produce event-free survival curves; differences between these curves were evaluated using a log-rank test. A multivariate analysis was performed using multivariate Cox proportional hazards regression analysis. For the selection of explanatory variables in the multivariate Cox regression analysis, the number of CAD events (n = 42) in this study was considered. In addition to pre-LpL mass, from the 14 factors that showed differences between group L and group H and/or with or without CAD event, five factors (i.e., pre-LpL mass, age, eGFR, hs-CRP, and skin AF) were ultimately adopted through stepwise method, clinical weighting, and consideration of multicollinearity. However, cut-off value of four factors (i.e., age, eGFR, hs-CRP, and skin AF) were evaluated using the receiver operating characteristic curves via the Youden index. Statistical significance was set at P values < 0.05.

## Results

### Baseline clinical characteristics

The baseline clinical characteristics of the studied groups are shown in Table 1. The mean pre-LpL mass for groups L and H was 46 ng/mL and 73 ng/mL, respectively. Group L was significantly associated with a higher frequency of male sex, being a smoker, and diabetes mellitus. Regarding biomarkers, serum triglyceride concentration, serum non-high-density lipoprotein cholesterol (non-HDL-C) concentration, fasting blood glucose concentration, hs-CRP, TyG index, frequency of albuminuria, and skin AF were significantly higher in group L than in group H. However, group L had a significantly lower eGFR, HDL cholesterol concentration, and frequency of RAS inhibitor and statin use than group H.

**Table 1:** Clinical Characteristics of the Studied Groups

Characteristics	Overall	Group H	Group L	P value
N (male/female)	480 (160/320)	269 (75/194)	211 (85/126)	0.004
Age (years)	75 ± 12	75 ± 12	76 ± 12	0.582
Serum LpL (ng/mL)	61 ± 17	73 ± 12	46 ± 8	< 0.001
Risk factors				
BMI (kg/m^2^)	23.1 ± 3.6	23.1 ± 3.5	23.1 ± 3.6	0.876
Smoker, n (%)	83 (17)	36 (13)	47 (22)	0.011
Hypertension, n (%)	338 (70)	190 (71)	148 (70)	0.907
SBP (mm Hg)	137 ± 15	137 ± 15	137 ± 16	0.971
DBP (mm Hg)	84 ± 11	84 ± 11	85 ± 11	0.663
Dyslipidemia, n (%)	334 (70)	189 (70)	145 (69)	0.717
Diabetes mellitus, n (%)	162 (34)	76 (28)	86 (41)	0.004
Clinical parameters				
eGFR (mL/min/1.73 m^2^)	47 ± 9	47 ± 9	46 ± 9	0.075
LDL cholesterol (mg/dL)	134 ± 35	132 ± 32	138 ± 38	0.113
Triglyceride (mg/dL)	130 ± 66	120 ± 63	143 ± 68	0.001
HDL cholesterol (mg/dL)	59 ± 15	63 ± 15	54 ± 13	< 0.001
Non-HDL cholesterol (mg/dL)	160 ± 37	156 ± 33	166 ± 41	0.004
FBG (mg/dL)	112 ± 22	109 ± 21	116 ± 23	0.007
hs-CRP (mg/L)	0.6 (0.2–1.2)	0.5 (0.1–0.8)	0.9 (0.4–1.7)	< 0.001
TyG index	8.8 ± 0.5	8.6 ± 0.6	8.9 ± 0.5	< 0.001
Albuminuria, n (%)	148 (31)	72 (27)	76 (36)	0.029
Skin AF (AU)	2.8 ± 0.5	2.6 ± 0.5	2.9 ± 0.5	0.001
Medication				
RAS inhibitor, n (%)	217 (45)	133 (49)	84 (40)	0.035
Statin, n (%)	199 (42)	123 (46)	76 (36)	0.032

Continuous values are presented as mean ± SD or median (25th–75th percentile). LpL: lipoprotein lipase; BMI: body mass index; SBP: systolic blood pressure; DBP: diastolic blood pressure; eGFR: estimated glomerular filtration rate; LDL: low-density lipoprotein; HDL: high-density lipoprotein; FBG: fasting blood glucose; hs-CRP: high-sensitivity C-reactive protein; TyG: triglyceride-glucose; AF: autofluorescence; AU: arbitrary unit; RAS: renin-angiotensin system.

### Kaplan–Meier curve analysis

**Figure 2:**
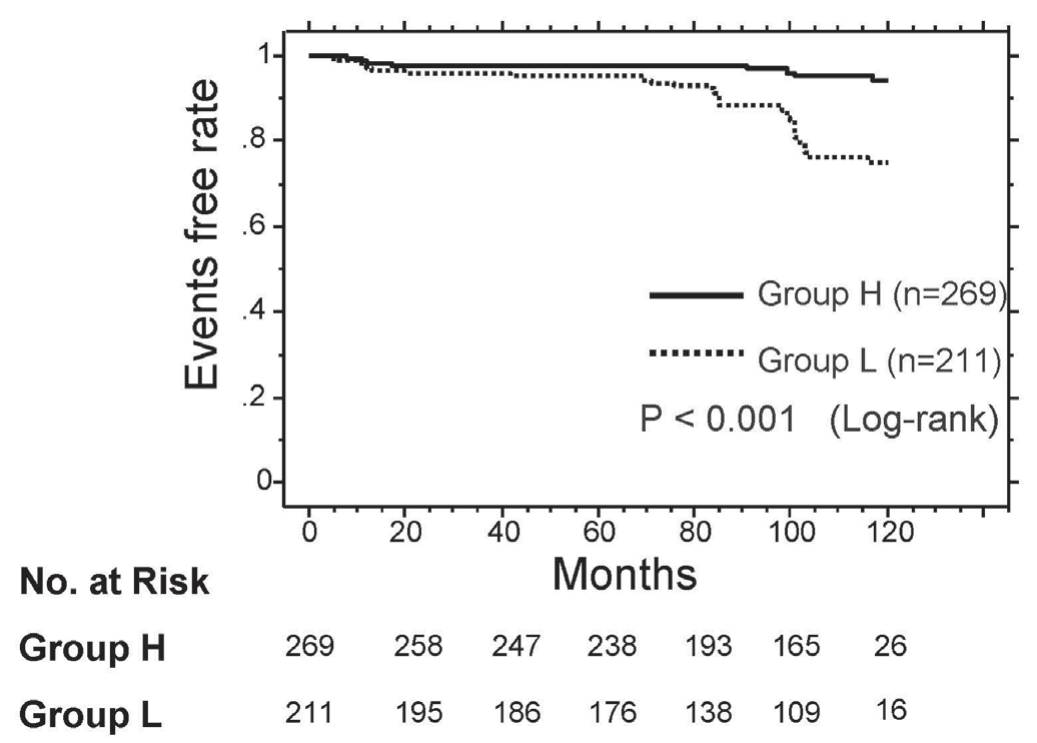
Kaplan–Meier curve analysis for the incidence of CAD event. During the observation period, group L (low pre-LpL mass) had a significantly higher incidence of a CAD event than group H (high pre-LpL mass, P < 0.001, log-rank test). CAD: coronary artery disease; pre-LpL mass: preheparin serum lipoprotein lipase mass.

The Kaplan–Meier curve analysis for the incidence of a primary CAD event is shown in Figure 2. The median observation period was 107 months (range: 2–120 months). During the observation period, a primary CAD event occurred in 42 patients (group L: n = 31 (14.7%) vs. group H: n = 11 (4.1%)). Group L had a significantly higher incidence of a primary CAD event than group H (P < 0.001, log-rank test).

### Association between the presence or absence of a primary CAD event and clinical parameters

Table 2 presents the association between the presence or absence of a primary CAD event and various clinical parameters. Age, frequency of diabetes mellitus, serum triglyceride concentration, non-HDL-C, hs-CRP, TyG index, frequency of albuminuria, and skin AF were significantly higher in patients who experienced a primary CAD event compared with those who did not. However, patients who experienced a primary CAD event had significantly lower eGFR, HDL-C, and RAS inhibitor and statin use than those who did not.

**Table 2:** Characteristics at Registration of Patients With and Without CAD Event

Characteristics	CAD event (–)	CAD event (+)	P value
N (male/female)	438 (142/296)	42 (18/24)	0.171
Age (years)	75 ± 12	80 ± 10	0.009
BMI (kg/m^2^)	23.1 ± 3.5	23.3 ± 4.1	0.697
Smoker, n (%)	74 (17)	9 (21)	0.459
Hypertension, n (%)	306 (70)	32 (76)	0.392
SBP (mm Hg)	137 ± 16	141 ± 12	0.112
DBP (mm Hg)	85 ± 11	83 ± 11	0.382
Dyslipidemia, n (%)	307 (70)	27 (64)	0.436
Diabetes mellitus, n (%)	140 (30)	22 (52)	0.007
eGFR (mL/min/1.73 m^2^)	47 ± 9	40 ± 9	< 0.001
LDL cholesterol (mg/dL)	133 ± 35	144 ± 36	0.059
Triglyceride (mg/dL)	128 ± 68	152 ± 44	0.026
HDL cholesterol (mg/dL)	59 ± 15	58 ± 11	0.528
Non-HDL cholesterol (mg/dL)	159 ± 37	174 ± 38	0.011
FBG (mg/dL)	112 ± 21	116 ± 25	0.307
hs-CRP (mg/L)	0.6 (0.2–1.2)	1.2 (0.5–1.3)	< 0.001
TyG index	8.7 ± 0.5	9.0 ± 0.3	< 0.001
Albuminuria n (%)	129 (30)	19 (45)	0.034
Skin AF (AU)	2.7 ± 0.5	3.1 ± 0.5	< 0.001
RAS inhibitor, n (%)	205 (47)	12 (29)	0.023
Statin, n (%)	191 (44)	8 (19)	0.002

Continuous values are presented as mean ± SD or median (25th–75th percentile). CAD: coronary artery disease; BMI: body mass index; SBP: systolic blood pressure; DBP: diastolic blood pressure; eGFR: estimated glomerular filtration rate; LDL: low-density lipoprotein; HDL: high-density lipoprotein; FBG: fasting blood glucose; hs-CRP: high sensitivity C reactive protein; TyG: triglyceride-glucose; AF: autofluorescence; AU: arbitrary unit; RAS: renin-angiotensin system.

### Multivariate Cox regression analysis

The results of multivariate Cox regression analysis for a primary CAD event are presented in Table 3. Among five factors, four factors (i.e., skin AF, pre-LpL mass, age, and hs-CRP) exhibited a significant hazard ratio for a primary CAD event. However, eGFR was not selected as significant hazard ratio for a primary CAD event.

**Table 3:** Multivariate Cox Regression Analysis for CAD Event

Variable	HR	95% CI	P value
Skin AF (≥ 2.8 AU)	2.96	1.53–5.75	0.001
Group L (vs. group H)	2.8	1.39–5.64	0.003
Age (≥ 80 years)	2.45	1.35–3.91	0.004
hs-CRP (≥ 1 mg/L)	2.17	1.19–3.17	0.011
eGFR (≤ 46)	2.11	0.88–5.04	0.092

CAD: coronary artery disease; HR: hazard ratio; CI: confidence interval; AF: autofluorescence; AU: arbitrary unit; hs-CRP: high-sensitivity C-reactive protein; eGFR: estimated glomerular filtration rate.

## Discussion

This prospective study was designed to investigate the clinical utility of the pre-LpL mass as a predictor for the primary CAD event in patients with CKD. Consistent with previous reports, in this study, pre-LpL mass of cut-off value to predict the primary CAD event was lower in male compared to female. The low pre-LpL mass group significantly developed more primary CAD events than the high pre-LpL mass group during the observation period, even though the composite CAD endpoint combines heterogeneous outcomes (revascularization, angina treatment, myocardial infarction), which may differ in pathophysiology and clinical significance. Furthermore, in the multiple Cox proportional hazards analysis, low pre-LpL mass was a significant predictor of the primary CAD event. However, skin AF, an *in vivo* indicator of AGEs, and hs-CRP, an index of inflammation, were also selected as significant primary CAD event predictors. In addition, a significant association was found between the pre-LpL mass and these two biomarkers at baseline analysis. Regarding the interests of this study, in the target population, aside from aging, conventional traditional coronary risk factors were not significant predictors of CAD onset. The study’s single-center nature limits generalizability. In addition, only 42 CAD events occurred, raising concerns about overfitting in the multivariate Cox regression model, and the predominance of female participants (66.7%) may affect external validity. However, the results of this study support the importance of low pre-LpL mass as a predictor of CAD event in patients with CKD.

### Significance of the pre-LpL mass as a CAD risk factor in CKD

Tsutsumi et al reported that the long-term administration of NO-1886, a drug that activates LpL, suppressed the progression of coronary arteriosclerotic lesions along with an increase in LpL in rat experiments [27]. Therefore, considering the animal experiments conducted by Tsutsumi et al [27] and the results of this study on the human body, it is believed that LpL may suppress the progression of coronary arteriosclerotic lesions in patients with CKD. Moreover, it is assumed that a decrease in the pre-LpL mass may be one of the factors that predicted the primary CAD event in patients with CKD. However, Tsutsumi et al [27] described that the role of HDL-C in reducing LpL is important for the suppression of coronary arteriosclerotic lesion progression. In the study results, the HDL-C in patients with a low pre-LpL mass was significantly lower than the HDL-C in high pre-LpL mass cases. In addition, the HDL-C in patients with primary CAD events was significantly lower than in cases with no primary CAD event; however, it was not selected as a factor in the multiple Cox proportional hazards analysis. Non-HDL-C also indicates the presence of an atherogenic lipoprotein such as remnant and other atherogenic lipoproteins [28]. Furthermore, several researchers have revealed a significant relationship between non-HDL-C and CAD [29, 30]. In the study results, the non-HDL-C concentration of the low pre-LpL mass group was significantly higher than that of the high pre-LpL mass group, and the non-HDL-C concentration was significantly higher in patients who experienced a primary CAD event than in those who did not. However, in this study, non-HDL-C was also not considered as a factor in the multiple Cox proportional hazards analysis. Certainly, a decrease in blood HDL-C concentrations and the appearance of remnants and other atherogenic lipoproteins are common findings of blood dyslipidemia in patients with CKD [31]. However, the results of this study indicated that reduction in pre-LpL mass behind these dyslipidemias appears to be more important in predicting the primary CAD event in patients with CKD.

It has been revealed by a number of studies that a decrease in eGFR significantly increases the occurrence of cardiovascular events in CKD patients [32, 33]. In this study, the eGFR of cases with primary CAD event was significantly lower compared to non-occurrence cases. In addition, the eGFR of cases with low pre-LpL mass was significantly lower than that of cases with high pre-LpL mass. However, the results of the multivariate Cox regression analysis did not select decrease in eGFR as a significant predictor of the primary CAD event. Although the importance of decreased eGFR in the development of cardiovascular diseases, including CAD event, is obvious, it is possible that the statistical significance of eGFR could not be detected due to the sample size of this study. However, the results of this study can be interpreted as that the decrease in pre-LpL mass may be a more important factor than decreased eGFR in the occurrence of the primary CAD event in patients with CKD. In the future, it is hoped that the validity of this study will be evaluated in larger-scale investigations.

### Insulin resistance and pre-LpL mass

Many researchers have emphasized the importance of insulin resistance in the development of CAD [34, 35]. In addition, Kobayashi et al reported that insulin resistance was independently associated with coronary artery calcification scores in patients with CKD [36]. However, several indices that clinically evaluate insulin resistance exist. Among them, the TyG index is a simple index calculated from fasting serum triglyceride and fasting blood glucose concentration. In addition, recent clinical studies have reported a significant association between the TyG index and CAD [37, 38]. In the results of this study, the TyG index of the group with CAD events was significantly higher than that of the group without a primary CAD event. However, the TyG index was not selected as a factor in the multiple Cox proportional hazards analysis in this study. However, in the univariate analysis, a significant association was found between the pre-LpL mass and TyG index. The pre-LpL mass has also been reported to be a useful indicator of insulin resistance [12, 25, 39]. Therefore, although both the TyG index and pre-LpL mass are useful indicators of insulin resistance clinically, the results of this study suggest that the pre-LpL mass is superior to the TyG index as a predictor of a primary CAD event in patients with CKD.

### AGEs, pre-LpL mass, and CAD

Basic and clinical studies have demonstrated that AGEs play an important role in the progression of coronary arteriosclerosis [40–42]. However, it has been pathologically confirmed that skin AF shows AGEs accumulation in the forearm [43]. Moreover, Hofmann et al reported that skin AF is associated with the presence of AGEs in the heart [44]. In fact, several clinical studies have shown that skin AF is significantly associated with coronary arteriosclerosis [45–47]. In this study, skin AF is the strongest predictor of a primary CAD event, which supports AGEs acts an important role for progression of coronary arteriosclerosis in patients with CKD. However, in the univariate analysis, the low pre-LpL mass group showed a significantly higher skin AF than the high pre-LpL mass group. It is suggested that LpL may act suppressively on AGEs, or AGEs may reduce LpL activity. However, limited literature exists regarding the relationship between LpL and AGEs in coronary arteriosclerotic lesions. Beauchamp et al reported that AGEs promoted LpL expression in macrophages [48]. This result contradicts the results of this study, which showed a negative correlation between the two biomarkers. Therefore, considering the results presented by Beauchamp et al [48], together with the results of this study, the relationships between pre-LpL and skin AF does not reflected relation of these two biomarkers in macrophages in coronary arteriosclerotic lesions, and it is speculated that the association of the two biomarkers in the coronary arterial vessel wall is observed in sites other than macrophages. However, there is a clear lack of basic and clinical evidence showing the relationship between LpL and AGEs in CAD, and it is hoped that future studies will clarify the association between these two biomarkers with a focus on coronary atherosclerotic lesions.

### Inflammation, pre-LpL mass, and CAD

Certainly, inflammation plays an important role in the mechanism of coronary arteriosclerotic lesion progression [49, 50]. In addition, researchers have reported the link between inflammation and coronary arteriosclerosis in CKD [51, 52]. In this study, hs-CRP as an inflammation marker was selected as an independent predictor of initial CAD events in CKD patients, although by itself it may not fully capture the inflammatory burden. However, in the univariate analysis, hs-CRP in the low pre-LpL mass group was significantly higher than that in the high pre-LpL mass group. Therefore, this result indicates that inflammation and a decrease in pre-LpL mass are clinically correlated. Several researchers have shown that RAS inhibitor and statin reduce inflammation in the coronary arterial vessel wall [53, 54]. However, clinical studies have reported that these drugs significantly increase the pre-LpL mass [55–58]. In this study, the rate of RAS inhibitor and statin use in the low pre-LpL mass group was significantly lower than that in the high pre-LpL mass group. Furthermore, in this study, RAS inhibitors and statins were not selected as factors in the multivariate Cox proportional hazards regression analysis due to the limited number of explanatory variables relative to the number of CAD events. However, the incidence of CAD events was significantly higher in patients who were not taking these medications compared to those who were. Therefore, the active use of these drugs in patients with a low pre-LpL mass is expected to prevent a primary CAD event in patients with CKD by decreasing inflammation and increasing the pre-LpL mass.

### Limitations

This study has several limitations. First, this study has shown several possible mechanisms of association between the pre-LpL mass and CAD in patients with CKD; however, the mechanisms have not been fully elucidated. Therefore, using basic and clinical methods, it is desirable to clarify the significance of the pre-LpL mass or LpL in coronary arteriosclerosis in CKD from various perspectives. Second, exclusion of patients without complete pre-LpL data may introduce selection bias. Third, there are substantial baseline differences (e.g., diabetes prevalence, medication use) between groups that may confound outcomes despite multivariate adjustment. Fourth, the use of a Japanese-specific eGFR equation limits applicability to other populations and should be acknowledged. In addition, the lack of significance of eGFR in multivariate analysis contradicts established literature and warrants a more cautious interpretation. Finally, while pre-LpL mass appears promising, the conclusions may overstate its utility as a predictor without external validation or intervention data. In addition, pre-LpL mass was measured only once at baseline, which limits conclusions about causality and longitudinal changes. Therefore, large, multicenter prospective trials are warranted to determine whether variations in the pre-LpL mass contribute to the development of a primary CAD event in patients with CKD, including interventional trials of RAS inhibitor and statin use.

### Conclusions

This prospective study showed that a decrease in the pre-LpL mass is a useful predictor of a primary CAD event in patients with CKD. Furthermore, background factors such as an increase in the AGEs and inflammation were also important factors in these patients. Further basic and clinical studies, including prospective clinical trials, are expected to be conducted to verify the detailed mechanism of the results of this study, and to determine whether interventions—such as RAS inhibitor and statin use—can reduce the incidence of primary CAD events in patients with CKD who have low pre-LpL mass.

## Data Availability

The author declares that data supporting the findings of this study are available within the article.

## Abbreviations

AF: autofluorescence
AGEs: advanced glycation end products
CAD: coronary artery disease
CKD: chronic kidney disease
CAVI: cardio-ankle vascular index
eGFR: estimated glomerular filtration rate
hs-CRP: high-sensitivity C-reactive protein
LpL: lipoprotein lipase
non-HDL-C: non-high-density lipoprotein cholesterol
pre-LpL mass: preheparin serum lipoprotein lipase mass
RAS: renin–angiotensin system
TyG: triglyceride-glucose
